# Supplementary material for: Experience of Cannabis Use from Adolescence to Adulthood in France: An Interpretative Phenomenological Analysis
Source: Int J Environ Res Public Health. 2023 Mar 2;20(5):4462. doi: 10.3390/ijerph20054462 (PMC10002113; doi:10.3390/ijerph20054462)
Supplement: Supplementary file 1 [file ijerph-20-04462-s001.zip › ijerph-2219383-supplementary.pdf]

Supplementary material

Log book

The Theme: Cannabis

Topic: Description of the experience of cannabis use among adults and older people: a qualitative approach

Issue: To gain an in-depth understanding of the motivations for using or stopping cannabis use in adulthood, to identify participants' beliefs, opinions and attitudes towards the product, and to explore participants' relationship with the health care system

Question: Why and how do adults and older people experience self-medication with cannabis rather than the use of medication?

Answers to the 7 questions that will be the focus of my diary:

1- What is my initial question?

What are the links between the socio-familial situation and the trajectories of cannabis use from adolescence to adulthood? How does using cannabis improve health? What is their relationship to cannabis?

2- How did I come to ask myself this question?

This question was suggested to me by my internship supervisors, but I have already asked myself this question in my medical practice.

Cardiology internship at Foch in Paris: meeting with a patient in his forties who had suffered from a heart attack. The next day, he asked for "a medicine to sleep" because he usually took cannabis. Father of a family who seemed anxious but I had no idea he was taking it, it surprised me.

3- If I were questioned myself, what would my answer be?

I've tried everything as far as medication is concerned, I'm in pain and nothing helps. But, guilt for doing something illegal. (In all honesty, I don't think I would have used cannabis even if no medicine worked→ but I haven't experienced such pain? /I am prejudiced about cannabis because of its illegality? The risk of addiction?)

4-Why am I convinced that this question is relevant?

It is a topical issue. Since March 26, 2021, France has started experimenting with cannabis for therapeutic purposes. It is important to understand in depth the reason for cannabis use, especially among the 30-40 years old, a population that is little mentioned in cannabis studies compared to younger people.

Major implication for public health.

5- What answers do I expect from the participants?

Cannabis use for purely medical reasons (physical health): because they are in pain, have a chronic illness, depression, anxiety... and conventional treatments have failed or else: Complicated family life, unfavorable socio-economic conditions, despair... push them to use cannabis.

6- What answers do I not expect from the participants?

I use cannabis only to feel good, because I am bored. (Answer generally given by the youngest) -->a priori to be deconstructed

7- What is finally my research question?

Why use cannabis for self-medication rather than for medication? How do the interviewees perceive cannabis use in relation to their environment/society? What are their cannabis use trajectories? Is this cannabis use purely "therapeutic"?

01/02/2022: 1st interview 14:30

A little apprehensive that it won't last long enough.
I imagine a blonde, smiling, single woman working at the post office who smokes during the afternoons, who consumes to "please" herself.
Afraid that she won't respond to reminders.
I hope it will go well.
I hope I'll be natural enough.
Post interview 16:52
Very nice woman. She doesn’t connect after 15 minutes. I decid to call her. She forgot about the interview and was going to take a nap. She coughs a bit on the phone. She says she's happy to answer questions if that helps. Her child is still sleeping, we have time. Small town not far from Marseille. Has a southern accent. The older one has Covid, 15 years old, she knows how to use zoom (for school). Computer too slow, connects to her phone. I would have liked to have been born in 2022 (zoom application asks for the date of birth). We wouldn't have been able to chat. She's laughing. She seems like a very sunny lady. She says "I have time" (this is mostly for you). Talks out loud while trying to get her computer to work.
Start of the interview on video:
Pretty redhead, with fringes (a bit short), hair tied back. Smiling. She was wearing a dark blue plaid jumper (fashionable, influencers on Instagram advertise it as "sweet plaid"). Took it off after a minute. Wearing a military green T-shirt. Hollow cheeks, rather thin, surrounded, very smiling and pleasant. Already confident.
She is not wearing headphones and the sound is on speaker when she talks to me
A dog passes behind her.
At first, she is in the living room, then in her room (small blackboard with a red heart).
Afraid of the judgement of others, "the doctors themselves..." (I did well not to introduce myself as a doctor).
Sunny house/ black and red kitchen and living room décor.
To explain where her pain is, shows her belly with her phone.
Moves when mother-in-law and partner arrive (blonde mother-in-law, partner has a beard, slightly overweight).
Mutes her microphone, I can hear a little annoyance.
Told them she was in a meeting, a meeting about what? / She hasn't worked for 3 years
Two children, two years old with a dummy.
Her 15 years old daughter covid+, wears a black mask, a white short and a black skirt.
Woman a bit depressed, bored, consumes because it's a need. Covid has turned her life upside down. Very affected by her parents' divorce. Would like to be a good mother +++ Has trouble dealing with all this... Kind, helpful woman, a bit lost. Considers herself an addict/ would not like her daughter to use.
Suggests a meeting with her daughter.
Insists that her use is now therapeutic whereas when she was young, recreational+++.
Made me feel a bit sorry. Needs to talk. Hides her sadness behind a smile (when she talks about her parents' divorce).

08/02/2022: 2nd interview 17:05 Cancelled
I feel a bit stressed, less than for the first interview but still a bit apprehensive
He's a man, maybe he'll be less "talkative"?
I wonder what he does for a living/ I imagine he works in a company (when I called him last week, he said he was working)
I hope that the interview will be as rich as the first one and that the person will open up

Interview postponed to 09/02 18:00
At first, he doesn't answer my call. I leave a message. He calls me back and explains that he is stuck at work. He hasn't checked his emails. Interview rescheduled for 6pm the next day. I'm a bit annoyed -> waste of time
09/02 no answer to my calls/messages.

14/02/2022: 2nd interview 10am
The lady on the phone sounds very nice. She has a deep voice (of someone who smokes a lot). She insists on anonymity. She says she is looking for a job so she can do the interview at any time.
I imagine that she is single, without children and that she uses cannabis to please herself, to take her mind off things.
It's February 14th (Valentine's Day), could this day be a bias and change her answers-> more or less "depressed" than another day X.
Post interview 12:05
An emotional challenging interview.
She tells me about things that are very difficult for her, including her relationships, sexual relationships, her rape at 17 (where she makes herself feel guilty and puts the blame partly on herself)
Her rape story touched me, without me letting anything show
She has tears in her eyes, her hair gathered up a bit, her cheeks red.
She talks about her self-image, her self-esteem, her overweight, her difficulty in finding a job
At the end of the interview, I try to discuss again and reassure her because I think it was emotionally hard for her (hypersensitive woman).

14/02/2022: 3rd interview 14:30
I imagine a father who has stopped using for his child
Post interview 14:53
He is a man with medium-length hair, no beard and a moustache, a bit of an artist. He tells me he is 1.93m tall. He speaks quickly and does not look me in the eye when he speaks about his consumption. He is a man of a certain socio-economic level. He is a smoker and seems a bit nervous, maybe that's his nature. Feels very guilty.
26/02/2022: 4th interview 16:55
Saturday afternoon. She was not very pleasant the first time on the phone but agreed to participate in the interview. I hope she will. I wonder if she has a heavy or complicated past to carry.
18:30 post interview:
Shy young woman. Studied biology and did a thesis which she stopped. She changed her career to marketing. Currently working at Roche in Boulogne. She is actually in Normandy with her parents. Has difficulty talking about herself. Seems very prone to depression. Cried. Did not dare to put the camera in front of her. Lying in bed during the interview. White wool jumper. A certain casualness that masks a great shyness+++.

07/03/2022: 5th interview 12:55
I'm quite serene. The person on the phone sounded serious and organised. I wonder how the interview will go.
14:25 post interview

The person had a peculiar face: a prominent chin. He tells me during the interview that he has acromegaly. He used to be a drug dealer. This surprises me. At the beginning, he doesn't look me in the eye, then he gradually opens up. He talks a lot.

08/03/2022: 6th interview 09:15
I wait for the interview to start. It is Women's Day. The interview has already been postponed. He was a bit annoyed. I hope he won't be in a too bad mood; I feel like I'm starting to get used to this kind of interview.
11:34 post interview.
He is a slightly fat man with a shaved head. He wears black rectangular glasses. He is a family man. He had a rather "complicated" youth. He was violent but is now coming to terms with it. He is now a father. He insists on the Maghrebian/Algerian side, and is curious to know more about me. I have the impression that he has understood my origins. His wife is Algerian. I was a bit apprehensive about him being silent. In the end, he spoke quite a bit.

18/03/2022: 7th interview 08:55
She is a woman who seems serious and very willing to participate to the interview. She sent her consent a week before the interview.
10:23 post interview.
She is a beautiful young woman. She was without her kitchen which overlooks a small garden. She was wearing a jogging suit. She looks older than her face, it must be her illness. She is sweet and looks like she has suffered from her illness. She seems brave.

04/04/2022: 8th interview 13:05
She works abroad. She replied to my email very quickly and seems very willing. She said she would be 15 to 30 minutes late. It's been a while since I've done any interviews.
15:00 post interview.
She cried in the first 5 minutes. She talked about a miscarriage. Her distress touched me. She cried several times. She wore a turban on her head. She looked older than her age. Her cheeks were hollow and streaked with tears. She was smoking an electronic cigarette while talking to me.

25/04/2022: 9th interview 09:49
She is a very nice woman on the phone, very friendly, who seems to be involved in research. By re-reading the characteristics of the quantitative study, I wonder if she does not really consume purely for recreational purposes.
11:20 post interview.
She is a very thin woman with greying hair. She is wearing a red turtleneck and a colorful striped jumper. She is very friendly and smiling.

20/05/2022: 10th interview 11.20am post interview
He talked about his wife. His wife was in the background, she goes behind him. I wonder if he may have censored himself at times. He didn't look me in the eye.

23/05/2022: 11th interview 11:35am post interview

He is a thin man with long grey hair tied back. He is in a kind of small office (he works from home), quite dark. There is a picture of a little girl carrying balloons. There is a background noise because of his headphones.

07/06/2022: 12th interview 17:35
I call her. She is still at work and asks me to reschedule the zoom meeting. So, we moved it to 6.30pm. (She had already offered me an interview and did not honor it)
20:00 post interview.
She was a full-figured woman. She didn't put the camera right in front of her but you could see the top of her face. She is nice and pleasant. There is her daughter in a room next door.
